# Beyond the Finish Line: The Impact and Dynamics of Biomarkers in Physical Exercise—A Narrative Review

**DOI:** 10.3390/jcm10214978

**Published:** 2021-10-27

**Authors:** Alexandru-Dan Costache, Irina-Iuliana Costache, Radu-Ștefan Miftode, Celina-Silvia Stafie, Maria-Magdalena Leon-Constantin, Mihai Roca, Andrei Drugescu, Delia-Melania Popa, Ovidiu Mitu, Ivona Mitu, Larisa-Ionela Miftode, Dan Iliescu, Cezar Honceriu, Florin Mitu

**Affiliations:** 1 Department of Cardiovascular Rehabilitation, Faculty of Medicine, University of Medicine and Pharmacy “Gr. T. Popa”, 700115 Iasi, Romania; adcostache@yahoo.com (A.-D.C.); leon_mariamagdalena@yahoo.com (M.-M.L.-C.); roca2m@yahoo.com (M.R.); andreidrugescu@yahoo.com (A.D.); mitu.florin@yahoo.com (F.M.); 2 Department of Internal Medicine I (Cardiology), Faculty of Medicine, University of Medicine and Pharmacy “Gr. T. Popa”, 700115 Iasi, Romania; ii.costache@yahoo.com (I.-I.C.); deliamelaniapopa@gmail.com (D.-M.P.); mituovidiu@yahoo.co.uk (O.M.); iliescud@gmail.com (D.I.); 3 Department of Preventive Medicine and Interdisciplinarity, Faculty of Medicine, University of Medicine and Pharmacy “Gr. T. Popa”, 700115 Iasi, Romania; celina.stafie@umfiasi.ro; 4 Department of Morpho-Functional Sciences II, Faculty of Medicine, University of Medicine and Pharmacy “Gr. T. Popa”, 700115 Iasi, Romania; ivonamitu@gmail.com; 5 Department of Infectious Diseases (Internal Medicine II), Faculty of Medicine, University of Medicine and Pharmacy “Gr. T. Popa”, 700115 Iasi, Romania; larisa.miftode@yahoo.com; 6 Faculty of Physical Education and Sports, “Alexandru Ioan Cuza” University, 700115 Iasi, Romania; chonceri@yahoo.fr

**Keywords:** physical exercise, immune system biomarkers, defensins

## Abstract

The research of biomarkers continues to emerge as a developing academic field which is attracting substantial interest. The study of biomarkers proves to be useful in developing and implementing new screening methods for a wide variety of diseases including in the sports area, whether for leisure activities or professional sports. Novel research has brought into question the immune system and the limitations it may impose on sports practicing. As the well-being of athletes is a priority, the state of their immune function offers valuable information regarding their health status and their ability to continue training. The assessment of various biomarkers may contribute to a more accurate risk stratification and subsequent prevention of some invalidating or even fatal pathologies such as the sudden cardiac death. Therefore, we have reviewed several studies that included sports-related pathology or specific morphofunctional alterations for which some immune biomarkers may represent an expression of the underlying mechanism. These include the defensins, immunoglobulin A (IgA), interleukin-6 (IL-6), the tumoral necrosis factor α (TNF-α), and the white blood cells (WBC) count. Similarly, also of significant interest are various endocrine biomarkers, such as cortisol and testosterone, as well as anabolic or catabolic markers, respectively. Literature data highlight that these values are greatly influenced not only by the duration, but also by the intensity of the physical exercise; moderate training sessions actually enhance the immune function of the body, while a significant increase in both duration and intensity of sports activity acts as a deleterious factor. Therefore, in this paper we aim to highlight the importance of biomarkers’ evaluation in connection with sports activities and a subsequent more adequate approach towards personalized training regimens.

## 1. Introduction

One of the main concerns in sports is related to the well-being of those who practice it. Whether we are talking about the individuals who practice leisure activities, or those who are professional players, certain risks are inherently present, ranging from small injuries to severe incapacitating pathologies, or even fatalities. Therefore, many studies are emphasizing the importance of new screening methods in order to detect individuals at risk, to prevent certain events, or to promote new and more efficient therapeutic approaches.

Physical exercise can be an inducer for the immune function, when performed with moderation [1]. However, when exaggerated, it can have an immunosuppressive effect, with a duration estimated between 3 to 72 h [2,3,4,5].

Even the same type of exercise can induce a rise in proinflammatory biomarkers when performed in high-intensity training sessions, whereas, when practiced at a lower intensity, it causes a decrease of the inflammatory status [6].

As increasing consideration is being given to prevention methods which may significantly improve the quality of life of these subjects and relieve the financial burden of the healthcare system, the immune system biomarkers could become a future standard screening method.

The immune system biomarkers’ variation in relation to the physical activity reflects how sports could stimulate the immune function of the body, thus preventing certain diseases, or, on the contrary, inhibit it and actually be a trigger factor in making the athletes more prone towards injuries and other invalidating conditions. Under these circumstances, we consider that a personalized approach based on a multimarker assessment (comprising either serum, salivary or skin biomarkers) combined with individual characteristics could potentially improve (or at least maintain) athletes’ health condition, avoiding inflammation-related injuries or underperformance.

Therefore, we reviewed data from newly-published studies which investigated the variations of defensins, Immunoglobulin A (IgA), white blood cells (WBC), interleukin-6 (IL-6), tumoral necrosis factor α (TNF-α), soluble tumor necrosis factor-like weak inducer of apoptosis (sTWEAK), soluble suppression of tumorigenicity-2 (sST2) and soluble cluster differentiation 163 (sCD163) as immune biomarkers. Additionally, we focused on the importance of various stress biomarkers such as cortisol, a catabolic marker, together with testosterone levels, an anabolic marker (Table 1).

## 2. Defensins

Human defensins are small antimicrobial peptides involved in infections and inflammation, presenting various protecting effects [7]. The two main classes are the alpha (α)-defensins, expressed by the neutrophils, which is why they are also called the human neutrophil peptides (HNP1-4) [8,9], or by the Paneth cells of the digestive or female reproductive systems, as is the case of the α-defensins 5 and 6 (HD5 and HD6) [10,11,12]. The other class is represented by the beta (β)-defensins (HBD1-4), which are mostly isolated from epithelial surfaces, such as the skin, oral, digestive and respiratory mucosae [13]. Both classes of defensins are highly involved in the innate immunity [14].

One of the main concerns in the medical field today, also involving athletes, is the rising prevalence of multi-drug resistant (MDR) upper respiratory tract infections (URTIs). These infections may induce severe and long-lasting complications that subsequently alter the physical capacity and the ability to perform or follow strict training regimens. Most often, the pathogens involved are *Streptococcus pneumoniae*, *Haemophilus influenzae*, *Staphylococcus aureus*, *Streptococcus pyogenes* and *Moraxella catarrhalis*. As the standard therapy involving the parenteral administration of beta-lactams and macrolides is becoming decreasingly efficient, and alternative therapies such as quinolones are being reserved for particular cases, new treatment methods are being researched, in order to optimize the management of such patients [15,16,17].

As with the afore-mentioned antibiotic-resistant URTIs, HBDs are not only involved in the specific modulation of the immune response, but may also represent a new therapeutic option when the classic antibiotics fail [18,19].

The levels of human defensins are in accordance with the duration and intensity of the physical exercise. Most studies showed an increased concentration in those who practiced sports, which further enhanced the immune system activity, regardless of the type of physical exercise [20,21,22,23,24].

However, when focusing upon these aspects, Eda et al. compared the secretion rates in two different types of sports (yoga versus cycling). While a HBD-2 increase was detected post-workout in both activities, it was also noticed that the immunity might and skin barrier might be depressed after high-intensity endurance exercises, with an enhanced risk of cutaneous infections. Therefore, one can conclude that only the moderate intensity exercises reflect an efficient immunological activity [22,23].

Age represents another important factor regarding the variations of salivary HBD-2 secretion. While the elderly tend to have a lower secretion of HBD-2, it may increase directly with the number of daily steps, up to a certain level, as an expression of a moderate form of daily exercise [25].

Concerning the long-term evolution of defensins levels during constant training sessions, HBD-1 and HNP1 levels steadily increased up to 6 months and 3 months, respectively. After reaching a maximum level, their concentration started to decrease, until a certain plateau threshold remained constant as long as the training schedule continued [26].

Summarizing the data above, one can notice that each of the conducted studies revealed an enhancement of the immune function in those who practiced sports by stimulating the defensin production. However, after a certain point, the risks outweigh the benefits due to the rise of the stress markers, which may alter the immune functions of the body [22,23].

Also, the body may reach an adaptive state at a certain point, as after long periods of steady training, with constant, stable, levels of both HNP1 and HBD-1 [26].

## 3. Immunoglobulin A

Immunoglobulins are a major group of proteins, being an essential part of the immune system [27,28]. The main representative of human antibodies is Immunoglobulin A (IgA), which is found in the saliva, tears or the intestinal mucosa, therefore being in the first-line defense of the mucosal humoral response. Its functions include the neutralization of toxins and the clearance of various pathogenic microorganisms, and it also plays an important role in the oral mucosal immunity by limiting its colonization and hence preventing the invasion of the epithelium, (e.g., against Candida albicans infections [29,30]). The beneficial effect of IgA at the level of the oral cavity is based on its enhanced inhibition of adhesion, reduction of hydrophobicity, and increased clearance of bacterial toxins. It is also considered an indirect biomarker of oral health, as the IgA salivary levels are higher amongst people with poor oral hygiene and multiple dental caries, acting as an index of immune surveillance in response to the accumulation of microorganisms [31,32].

IgA serum levels generally decrease post-workout compared to pre-workout, with a recovery time of 24 h. Consequently, this “immune gap” increases the susceptibility of developing URTIs [19,20,21,33,34].

Trochomiak et al. further detailed these findings in 2012 by adding that, although IgA production is decreased after training sessions, it may be stimulated during moderate intensity exercises, an aspect that is also confirmed by Eda et al. in 2018 [35,36].

However, this hypothesis is brought into question by Suzuki and Hayashida; in a 2021 article they acknowledge that high-intensity sports do have a depressing effect on the cell-mediated immunity, yet they maintain that although yoga or other low- and moderate-intensity exercises are not harmful, they do not stimulate in any way the cell-mediated immunity [37]. Moreover, the usefulness of IgA as a potential respiratory tract infection biomarker is being contested by Turner et al. in 2021, who, although acknowledging the results, cite a lack of reproducibility and low specificity and sensitivity in the detection of RTI susceptibility [38].

A similar statement was made by Sawczuk et al. in 2021, who concluded that the salivary IgA concentration and secretion rate are useful in assessing the short and long-term risks respectively for URTIs, yet none may predict the onset [39].

To sum up, although high intensity sports may act as an inhibitor for the immune system and increase the risk for respiratory infections, moderate intensity physical exercises may actually stimulate the defense mechanisms and help to prevent the occurrence of such pathologies.

## 4. Leukocytes

There is growing interest towards the so-called “exercise immunology”, more specifically how lymphocytes subsets are influenced by the type and intensity of sports training (intense versus moderate, professional versus amateur in different types of sport disciplines). One study highlighted an interesting aspect: athletes that follow a regular training pattern (without sudden variations or high-intensity competitions) during an entire season present a relatively constant cell count, with the exception of natural-killer (NK) cells [40]. The authors observed a significant increase in both NK total count and percentage, a somewhat different discovery from the previous classical concept that stressed the NK count decrease soon after an intense workout, an aspect linked with increased susceptibility to respiratory infections [41]. This latter concept is based on the observation that following an acute bout of physical exercise there is an enhanced proliferation of leukocytes, due to physical stress, followed by a consecutive drop in their count during the recovery period, sometimes referred to as the “open window of immunodepression”. Therefore, a complete blood count with differential (CBC/diff) could be an analysis of special interest in athletes, in order to evaluate the bidirectional relationship between the immune function response and the type or intensity of the physical training [42].

Neutrophil and monocyte values tend to increase after physical effort, while lymphocyte values have a biphasic response. Following a vigorous one-hour training, initially there is a marked lymphocytosis driven by a significant influx of NK cells, which rise by up to 10-fold, and by a 2.5-fold increased CD8+ T cells. This exercise-related mobilization is caused by peripheric mobilization due to muscle contraction and surge in arterial blood pressure. Another important mechanism is related to increased adrenergic stimulation (i.e., beta-2 adrenergic receptors on the lymphocytes) due to physical effort that enhances the release of lymphocytes into the bloodstream [43]. Following exercise cessation, the classic lymphocyte count dynamic is characterized by a dramatic decrease in numbers in the bloodstream, with a minimum level being recorded 1–2 h after the training session and a return to previous pre-exercise levels within 24 h. Although initially considered an “open-window” for opportunistic infections, a more modern viewpoint considers that this transient lymphopenia is actually beneficial, as it redeploys lymphocytes to peripheral tissues to conduct immune surveillance and subsequent clearance of injured, tumoral or pathogen-infected cells [43,44]. In fact, there is growing evidence claiming that physical activities stimulate the immune function and slow down the immunosenescence phenomenon [43]. In terms of the influence exerted by the type of exercise, a comparative study showed that continuous endurance training caused a decrease in lymphocyte count as opposed to high-intensity interval training, while all the other leukocyte fractions showed no significant variations [45].

However, there are also differences concerning single bouts of intense exercise compared to multiple, prolonged training sessions, as only short recovery periods between training sessions may actually exacerbate the immunosuppressed status [42].

Even when analyzed over a 12-month period, neither the WBC count, nor each individual type of leukocyte (neutrophils, eosinophils, basophils, lymphocytes and monocytes) showed any significant variation. This is also the case with regard to the adaptive state which ensues during a chronic exposure to physical stress, as there is a significant time interval between determinations in order to highlight the acute rise in WBC count [26].

## 5. The Endocrine Pathway: From Hormone Levels to Physical Performance and Viceversa

The assessment of energy availability is of utmost importance in sportsmen activity, needing to be evaluated both in routine daily training and during competitions or high-intensity training periods. The importance of measuring bioenergetic hormones in athletes in order to prevent the occurrence of some deleterious effects resulting from overtraining resides in the detection of the so-called chronic low energy availability (LEA) [46,47]. Of course, there are some simple methods to estimate energy availability, such as a dynamic monitoring of body mass or calculating the difference between energy intake and energy consumption in a certain period of time; however, these methods are often inaccurate, being biased by several factors [47].

In this context, the variation in serum levels of some endocrine biomarkers could prove useful in evaluating the available energy. Biochemically relevant decreases in serum levels of insulin-like growth factor-1 (IGF-1), cortisol, thyroid hormones, testosterone or ovarian hormones was observed in energy-deficient athletes [47,48,49,50]. Moreover, some of these biomarkers are also associated not only with a low-energy status, but also with significant morphological alterations which exert direct impact on physical performance. In this regard, it has been observed that IGF-1, a biomarker associated with adequate bone quality, decreases in female athletes with low bone mineral density and increased risk of stress fractures, which are a very common injury among women practicing sports, when compared to men [51]. Therefore, timely identification of bone demineralization via IGF-1 screening could prevent not only the potential occurrence of a stress fracture, but also a premature withdrawal from sports life.

Supporting the above statement, in male athletes a mutual relationship between hypogonadism and LEA was observed due to poor energy intake in periods with excessive training [52]. Furthermore, a similar pattern was found in females; the athletes with normal ovarian function (estradiol and progesterone levels) presented an improved sport performance compared to their counterparts with suppressed ovarian hormones and LEA [47,53]. This aspect draws attention towards the individualization of nutritional intake (both in terms of quantity and composition), these personalized regimens requiring to be correlated with the type, intensity and duration of the physical exercise, and to some other personal habits, such as comorbidities, sleep patterns or food allergies. Concerning the latter, Immunoglobulin E-based testing kits represent an adequate approach to assess suspected allergies to a specific food or even the individual’s unique metabolic feedback to certain complex nutritional products that contain a plethora of potential allergens and are very common among the athletes [54]. Basically, currently available IgE-based tests can predict allergies to specific foods with an accuracy greater than 95% [55].

Cortisol is an endogenous stress marker and may offer valuable information in relation to the immunosuppression which may appear in athletes after prolonged training sessions. Being a marker of the catabolic state of the body, it is useful to, in parallel, evaluate the testosterone levels, an anabolic marker, in order to calculate the cortisol/testosterone ratio and to assess whether the body is in an anabolic or catabolic state. Testosterone’s anabolic properties are explained through its inhibition of the transforming growth factor beta (TGF-β) signal, therefore reducing osteoprogenitor cells and increasing lean muscle mass. It also provides the necessary energy to the muscles and stimulates the reconstruction of glycogen reserves. The result is an increase in lean muscle mass and a reduction in fat muscle tissue [26,56,57].

In light of the fact that salivary cortisol levels rise during and after physical exercise [21], several studies also highlighted the inhibitory effect glucocorticoids have on salivary defensin secretion and concentration, this being an important deleterious corticoid-induced consequence on the immune system [58,59,60].

As Pero et al. showed in 2020, both cortisol and testosterone exhibited a similar pattern by constantly increasing their levels during a 6-months period before returning to the baseline values. This is a strong indicator of a systemic stress response and the adaptive modifications which occur simultaneously, before reaching a steady state [26].

These findings could be compared to those made by Kuoppasalmi et al. which stated a few decades ago that prolonged exercise induces a 30% reduction in the testosterone/cortisol ratio, yet Urhausen et al. more recently found that after a maximum aerobic training session, the testosterone levels had a major increase compared to that of cortisol, which presented only a slightly elevated serum level [61,62].

Once again, this proves the different impacts exerted by sport type on hormonal secretions, especially with regard to the aerobic versus anaerobic programs.

## 6. Interleukins and Tumor Necrosis Factor: More than Classical Inflammatory Biomarkers

Intensive exercise training (especially in professional athletes) is usually associated with various degrees of muscular injury, which can further trigger a variable immune response, with subsequent inflammation. A persistent, chronic inflammation can suggest either ongoing muscle damage, or an acute stress due to overtraining. Moreover, a detected serum peak of inflammatory molecules can also be a marker for an acute illness, such as respiratory tract infections, autoimmune conditions or even cardiovascular diseases [54]. Therefore, concurrent assessment of muscle injury biomarkers (e.g., creatin kinase) when determining inflammatory biomarkers in athletes is essential, in order to contextualize the etiology of inflammation and to define the subsequent approach, not only for an athlete’s particular sport performance, but ultimately for the athlete’s health status.

After the initial injury, as the inflammation develops, the WBC from the injury site (e.g., macrophages) shifts to anti-inflammatory profiles, thus releasing several cytokines and growth factors in order to limit the deleterious effects and to promote a tissue restoration. Interleukin-6 (IL-6), IL-1β, IL-10, IL-8 and the tumor necrosis factor-α (TNF-α) are markers of inflammation produced by the T-cells [63,64]. IL-6 is a dual pro-inflammatory and anti-inflammatory cytokine, involved in the C-reactive protein’s (CRP) expression, and, therefore, with an important role in the development of almost all chronic inflammatory diseases, but it also presents some beneficial, regenerative properties. In sports injury, especially in athletes who have a history of concussion, the balance is shifted towards pro-inflammatory effects, with significant perturbations in circulating levels of IL-6, therefore rendering it as a potentially useful biomarker in these situations [65,66,67].

There is no consensus about a general threshold concerning the serum levels of these inflammatory markers, the tendency being to individualize a “normal range”. This paradigm is based on repeated measurements, starting with a resting, healthy, baseline and continuing through different types of exercise or training intensities. Assessment of biomarkers during a competitional tournament or a period of heavy training can outline a physiological profile, in order to determine what are normal fluctuations and what are the variations associated with physical injury or other major health concerns [54]. Intermediate physical exercise down-regulates interleukin and TNF-α production, therefore reducing the inflammation. These findings are further confirmed by the results from a study conducted by Raguzzini et al. in 2021 on a simulated wheelchair basketball match, the increased IL-6 levels being positively correlated with the basal energy expenditure. However, the aforementioned study was correlated with the diet of athletes, the influence of which potentially represented a source of controversy [68].

Nevertheless, this scenario can be assumed as a screening tool for the regular assessment of certain biomarkers in athletes at high risk of injury. However, there is a lack of standardized protocols concerning what specific biomarkers should be evaluated for risk stratification. Rather, as we previously mentioned, an individualized approach is encouraged, the data in the literature emphasizing that a rise in serum levels of inflammatory biomarkers such as IL-6, IL-8, transforming growth factor β (TGF-β) or C-reactive protein (CRP) could be indicative of early, subclinical injury, hence allowing an adequate preventive approach, whether we are considering stopping physical exertion, additional diagnostic explorations or even emergency treatment [69,70]. Of note, the results should be contextualized with the biomarkers of muscle injury (i.e., creatine-kinase), the intensity of the training regimen or with other concomitant conditions (e.g., infections, anemia, dyslipidemia) that could alter the screening value of the inflammatory biomarkers [54]. An integrative screening approach should also include questionnaires that refer to subjective physical fatigue, sport-related mental stress (e.g., due to competitions, intense training sessions, financial burden) and even the evaluation of stress hormone levels, such as cortisol [54,71].

Despite the fact they are positively correlated with each other, some inflammatory biomarkers are negatively associated with the WBC and platelet count, which, in contrast, are elevated after training [72]. Therefore, in addition to these biomarkers, a CBC/diff may add certain value to the diagnosis approach. Although the CBC/diff assay is not itself a proper inflammatory marker, it provides significant information concerning a potential infection or describes the shifts in immune cell populations that characterize the inflammation induced by muscle damage [54].

## 7. The Soluble Tumor Necrosis Factor-Like Weak Inducer of Apoptosis, Soluble Cluster Differentiation 163 and sST2: Promising Perspectives

The tumor necrosis factor-like weak inducer of apoptosis (TWEAK) is expressed in multiple tissues, including human skeletal muscle and blood cells, with leukocytes constituting a major source of TWEAK and contributing to its role in inflammation-related injury [73]. Basically, TWEAK is a secreted pro-inflammatory multifunctional ligand of the TNF super-family, being involved in chronic inflammation and in the development of clinically-relevant associated processes, such as atherosclerosis [74,75]. This is explained by its binding to its initial receptor, the fibroblast growth factor inducible molecule 14 (FN14), therefore triggering an inflammatory response through IL-6 and IL-8 formation [76]. The TWEAK-FN14 axis further signals via the TNF receptors, thus activating the nuclear factor kappa-light-chain-enhancer of activated B cells (NF-κB) pathway, which results in an impaired myogenesis due to excessive myoblast proliferation [77]. Moreover, experimental data revealed that persistently elevated levels of TWEAK also seem to activate the classical NF-κB pathway, thus causing significant skeletal muscle atrophy, while, in contrast, decreased TWEAK concentrations up-regulate an alternative NF-κB pathway, prompting a myogenesis by stimulating myoblast fusion [77,78].

Another receptor for TWEAK is represented by the cluster differentiation 163 (CD163), which is also a scavenger receptor and a modulatory cytokine of inflammation. An increased CD 163 expression down-regulates TWEAK, thereby attenuating the proinflammatory status [79,80,81].

Regular physical activity causes a slight increase in both TWEAK and sCD163 levels, therefore maintaining an equilibrium between pro- and anti-inflammatory markers [81,82]. In this regard, literature data emphasizes that mild, temporary activation of the TWEAK-FN14 axis by regular physical activity is a physiological and promyogenic phenomenon, while a persistent chronic axis activation (due to injuries or hyperinflammation) induces increased proteolysis, thus causing subsequent muscular atrophy and sports underperformance [77,83].

Interestingly, a 2021 study conducted by Benedetti et al. on ultramarathon athletes following a 24-h race showed that TWEAK values decreased during effort, while sCD163 values increased in a positive relationship with IL-6 and the cardiac biomarkers troponin-I and NT-proBNP [84].

Exercise intolerance, increased fatigue and decreased physical performance are elements directly associated with heart failure (HF). A person who regularly practices sports activities will notice any limitations concerning their previous effort capacity, as this could be the first sign of an incipient, subclinical HF. However, there is a high degree of individual variability and subjectivism in the way patients feel and quantify these symptoms, this being a sufficient reason why there is an increased need for a validated method in detecting subclinical myocardial injury. Soluble suppression of tumorigenicity-2 (sST2) is a relatively new biomarker, with promising results concerning the assessment of both myocardial injury and inflammation [85]. High-intensity training increases myocardial stress due to tachycardia and increased blood pressure during exercise. Previous data showed that cardiac biomarkers such as troponins or natriuretic peptides are increased after prolonged and/or intense physical exercise, usually followed by a normalization of their serum levels within the next 24–72 h [86]. In the case of persistently elevated biomarkers in athletes, multiple mechanisms are incriminated, such as myocardial hypertrophy with subsequent fibrosis, chamber enlargement, oxidative stress or myocardial inflammation [87].

In the case of sST2, Aengevaeren et al. recently presented some very interesting results, by showing that marathon runners exhibited significantly increased sST2 concentrations (above cutoff values) both at baseline (48%) and after finish (94%). Moreover, the more rapid runners had higher sST2 concentrations. The same authors also found that the evaluated athletes had similar sST2 concentrations as patients with chronic or even acute heart failure at the start line and after the marathon, respectively [86,88]. sST2 is a proven predictor for mortality and poor outcome in HF [89,90,91], and therefore it is controversial that apparently healthy athletes express such high values of sST2. A reasonable explanatory hypothesis is that elevated sST2 concentrations in athletes may be induced by regular exposure to increased cardiac strain during exercise, as this may trigger the cardiac myocytes to release sST2 in circulation. On the other hand, regular exercise training may also elevate sST2 baseline values in certain athletes, whereas a very intense effort (e.g., marathon) may additionally increase sST2 concentrations due to rapid and sustained increases in cardiac load [86].

## 8. The Influence of Diet on the Immune System Biomarkers’ Variations: More than Muscle Fuel

Several results from the studies mentioned in this review are potentially influenced by different constitutional and environmental factors, such as the type of sport, the climate, the gender and, a very important factor, the diet.

Athletes who follow a ketogenic diet (KD) show a higher expression of multi-antigen-stimulated PBMC γ-interferon (IFN-γ) mRNA expression and a higher IFN-γ/IL-4 mRNA expression ratio, as well as higher multi-antigen-stimulated whole-blood IL-10 production and salivary immunoglobulin A (SIgA) secretion rate. IL-1β, IL-2, IL-8, and IFN-γ productions are lower in those who followed a habitual diet (HD) [92]. Also, intermittent fasting causes an increase in muscle damage biomarkers [93].

Active and constant physical training also influences the lipid profile by reducing levels of total cholesterol and triglycerides, thereby improving cardiovascular function with an indirect improvement in sports performance. More than just energy providers, some types of lipids play important roles in recovery after intensive training or even injuries. Omega-3 fatty acids eicosapentaenoic acid (EPA) and docosahexaenoic acid (DHA) are well-established protective factors against inflammation, also alleviating muscle fatigue or pain [54,94].

Another interesting aspect refers to the relationship between vitamin D status and inflammation in high-performance athletes. Willis et al. found a surprisingly high proportion of athletes with vitamin D deficiency, who also showed a direct association with increased serum levels of TNF-α. Low vitamin D and inflammation could represent the recipe for serious injuries, risk of infections and long-term impaired performance in athletes, therefore vitamin D supplementation may represent a reasonable approach in these cases [95].

In fact, nutritional status should be subject to periodic screening in athletes (even several times a year) in order to identify certain deficiencies (e.g., anemia, iron and vitamins deficiency) that can impair the physical performance and/or augment the risk of injuries [96]. The role of macronutrients, such as lipids, in athletes’ metabolism is well established, as the fats are the primary source of energy in endurance training or when there is a deficit of carbohydrates [54]. In the last decade, a special importance concerning “performance” diet is given to Omega-3 fatty acids for their positive impact in alleviating muscle soreness and reduction of muscle inflammation and perceived pain following intense training sessions [54,94].

However, in recent years the focus has shifted towards micronutrients, comprising minerals, vitamins or other various microelements exhibiting a plethora of beneficial effects. For example, vitamin D acts via various mechanisms; in addition to its classic role in maintaining bone integrity, vitamin D is involved in muscle metabolism, as several studies highlighted an enhanced aerobic performance in athletes who took vitamin D supplements [97,98]. Of similar importance in physical performance, we mention B complex vitamins, which influence the energy metabolism, or vitamin E, which acts as an antioxidant in cell membranes and other cellular structures, thus preventing muscle cell injury or anemia due to hemolysis [54].

Bringing antioxidant capacity into the discussion, it is essential to mention the increased oxidative damage that is associated with intense training sessions, as overexpression of intracellular aerobic reactions occur due to intense physical exercise, with a subsequent increased production of reactive oxygen species (ROS) [99]. As a result, the supplementation with various antioxidants could be an effective strategy in preventing muscle injury or even a more severe neuromuscular damage. The current trend is to emphasize the use of polyphenols, as these molecules have already shown beneficial effects in the amelioration of oxidative stress following intense training in athletes from a wide range of sport disciplines [100,101]. The polyphenols may act directly at the muscle level, via their functional hydroxyl groups that suppress the ROS synthesis, the enhanced chelation and clearance of microelements involved in ROS production, and via the scavenging of already existing ROS. The indirect pathway through which polyphenols could enhance physical performance is the alleged positive impact of certain plant extracts on myocardial function, effects mediated by their strong polyphenol-related antioxidant capacity, with promising results both in vitro [102,103] and in vivo [104,105].

## 9. Endurance vs. Strength: How to Choose Wisely

One of the main debates in the world of sports is in recent years focused on what type of exercises should be performed, more specifically, which one offers the best performance (in the case of professional players) or the most balanced ratio between physical condition and well-being. The two main categories are endurance (e.g., jogging) and strength (e.g., weightlifting).

The classical perspective gravitates towards the fact that endurance exercises will enhance muscle strength, doubled by improved oxygen delivery to the tissues, while strength-focused training will determine an increase in the muscle mass [106].

Nonetheless, endurance and strength exercise sessions can also be combined for both aerobic capacity and muscular mass development. It is however recommended to have an up to 24 h training-free interval between the strength and endurance sessions, in order for the adenosine monophosphate activated kinase’s (AMPK) activity to return to normal and to not interfere with the mammalian target of rapamycin (mTOR) signaling, which is also highly involved in resistance training adaptation [107].

When comparing the immunohematological profiles of endurance—(e.g., swimming) and power-oriented athletes (e.g., judo), the latter had lower monocytes and eosinophils but higher neutrophils percentages. They may also register decreases in hematocrit and CD16 values during the competitive season, while amongst endurance-oriented athletes one reported increases in hemoglobin and hematocrit levels. This could be explained by the microlesions which occur during combat sports that cause an erythrocyte reduction doubled by a stimulation of leukocytes production [108].

A study investigating the immunological responses (WBC and high sensitive C-reactive protein levels) to four different loading protocols (sauna, endurance + sauna, strength + sauna, endurance + strength + sauna), before, during, immediately after, 30 min after and 24 h after the training sessions, showed an increase in WBC in all groups during, immediately after and 30 min after the exercises; the high sensitive C-reactive protein levels registered no changes irrespective of measurement point or loading protocol. Also, concerning the acute hemodinamic response, the most significant drop in the systolic blood pressure values was registered following the endurance + sauna protocol, thus highlighting potential benefits in hypertensive athletes or aging leisure-oriented individuals [109].

Very important, exhaustive submaximal endurance and resistance exercises were shown to induce a surge in the systemic oxidative stress (through the rise of cortisol and intracellular ROS levels), with subsequent immunosupression (due to the decline in the CD4+ T cells to CD8+ T cells ratio) [110].

A study published in 2018 by Shaharudin et al. assigned a group of 19 weightlifters to perform either isotonic or isokinetic exercises, with blood samples taken at rest, immediately after completion and 1 month later. The results showed a decrease in total leucocyte count in both groups, which persisted, though, only in the isotonic group for 1 month [111]. Therefore, one can conclude that endurance exercises tend to cause a further decrease in the immune function, even though insignificant. However, in a more recent study, a combined strength-endurance training showed a significant reduction in senescence-prone T-cells, which was not observed in a comparable group who followed intensive strength training programs [112].

Concerning impact upon testosterone levels, Wade et al. showed that, although testosterone levels (both free and total), may drop following heavy exercise, no significant difference was observed between the isokinetic and isotonic practicing groups [113]. On the contrary, a recent study emphasizes the role of high-intensity interval training (HIIT) in the significant augmentation of both total and free testosterone compared to the baseline [114]. This aspect is of particular importance especially in aging sportsmen or in elderly who practice leisure sports and feel out of shape. The age-associated decreased testosterone synthesis leads to impaired cardiorespiratory performance, loss of muscle strength and poor general stamina [115,116], these deleterious consequences represent sufficient reasons to consider special training techniques such as HIIT, that improve the bioavailable testosterone. Hayes et al. found very interesting results; they observed an increase in total testosterone, but not in the free-testosterone in sedentary men following routine, moderate-intensity training. Free testosterone levels were boosted only after constant sessions of HIIT, the accepted paradigm being that middle-aged or elderly men should perform moderate exercise training alternating with regular HIIT sessions [117].

Another aspect which is sometimes overlooked is the manner in which certain exercises are performed. It has been proven that slow movement enhances the hormonal response, especially with regard to free testosterone and catecholamine levels, as compared to normal movement [118].

An important factor affecting the cardiovascular endurance is represented by the iron status. There is extensive evidence that links iron deficiency to impaired physical performance, because of its fundamental role in oxygen transport, oxidative phosphorylation and other various mechanisms, which are directly related to sports (Table 2). The professional athletes are particularly prone to iron deficiency due to a combination of common, real-life situations (Table 2) that compromise the optimal iron status, expressed as low serum iron, abnormally high total iron binding capacity, low transferrin saturation, and decreased levels of ferritin [54]. Basically, regular assessment of iron status before, during and after intense training sessions can be helpful not only for timely therapeutic management, but also for a customized diet, lifestyle or even types of training and their optimal schedule.

## 10. The Benefits of Physical Exercise: Good, but Not Perfect

As a general observation, in the initial stages or during moderate training sessions, the immune function is stimulated as noticed by the dynamic of the above-mentioned biomarkers, as well as the uniform growth of the muscle mass, especially with the help of the testosterone/cortisol ratio.

If these sessions are over-prolonged or too high in intensity, the catabolic processes will overwhelm the anabolic ones, therefore leading to degradation of the muscle mass and of the immune function.

Therefore, one can notice the double roles which these biomarkers play in monitoring athletes: early-identification of at-risk persons whose conditions may restrict them for further practicing performance sports and the assessment of the optimal training sessions for athletes during the competitive season.

It is important to mention that, although most of these findings focused on athletes, they are not restricted to them, as they could also be applied to those who practice sports leisurely.

However, when comparing these studies, it is important to take into account the different sports which have been evaluated and also the effects of the diet and other external factors such as the climate with regards to their effects on the body’s response.

All things considered, one should never feel deterred from starting and following a physical training schedule. Even though sports have certain risks including immunodepression or an increased inflammatory state or even more severe complications, these tend to appear only when practiced chaotically or excessively. If practiced in moderation, such risks are virtually non-existent, while the benefits are obvious and undeniable.

The beneficial effects of physical exercise are well-known and overall accepted within the scientific world. There are studies which acknowledge the anti-inflammatory inducing properties of sports, especially when it comes to the older population, and underline the importance of practicing a form of training program, even at more advanced ages [122].

Physical exercise is an important factor in preventing or in the recovery process of the coronary patient, by counteracting the onset and progression of atherosclerosis [123].

Exercise, associated with a supplementation of vitamin C, proved to be beneficial in reducing blood pressure value, along with down-regulating pro-inflammatory cytokines (PIC), in the gut mucosa, and reactive oxygen species (ROS), in the hypothalamus paraventricular nucleus (PVN), while up-regulating the anti-inflammatory cytokines (AIC) in the intestinal mucosa. Also, the intestinal microbiota showed significant improvement, through the up-regulation of deficient micro-organisms, such as Turicibacter and Romboutsia, involved in the production of short-chain fatty acids [124].

Considering how both asthma and obesity have an increasing prevalence world-wide, but also given their mutual relationship, one could easily see the impact physical exercise has in promoting weight loss and, therefore, asthma symptomatology reduction. Adipose tissue induces an increase in pro-inflammatory mediators’ production such as M1 macrophages, and adipokines (leptin), and a reduction in anti-inflammatory cytokines such as adiponectin [125].

Swimming, as a form of aerobic exercise did show a down-regulating effect on the inflammatory and destructive responses induced by high-fat diets (HFD) and alleviating insulin-resistance in mice, as compared to those which were only subjected to the HFD, without any form of physical exercise [126,127].

Age is a classic inducer of many alterations within the immune system, Kawanishi and Machida recently noticed a significantly increased number of neutrophils and Ly-6C+ inflammatory macrophages in the skeletal muscle of older mice, compared to younger counterparts, as well as augmented inflammation and oxidative stress, biochemically assessed using phosphorylated c-Jun N-terminal kinases (JNK) and nitrotyrosine [128]. In the elderly female population, physical training programs, whether associated or not with grape juice consumption, showed anti-inflammatory effects and a positive influence in the antioxidant defenses, both enzymatic and non-enzymatic, while those who followed a grape juice diet, without exercise, showed an increase only in non-enzymatic antioxidant defense [129]. An experimental study that approached the age-related influence of physical exercise found that 4-week treadmill exercise improved metabolic function and also increased the lean muscle mass, while decreasing the fat mass, in 88-week-old mice. This was also quantified using the senescence marker p16 in the white adipose tissue, with a significantly decreased expression following the training program [130].

In stroke patients, the status of the muscles tissue is important in the outcome of the recovery programs, with better results in patients with a more developed muscle mass. This is especially since inflammation is one the most adverse factors in stroke development and recovery [131].

Cognitive disorders may also be improved upon with physical exercise as a non-drug treatment schedule, as it showed in older adults, where it modulated stress and anti-inflammatory cytokines. This was quantified by measuring the levels of cortisol, IL-6, TNF-α, and CRP [132].

Intensive locomotor training (ILT) showed an improvement in pain control in rats who sustained spinal cord injuries (SCI), as compared to those who did not follow a training program, along with improved locomotor outcomes and prolonged survival. This may be due to the anti-inflammatory effect and the stimulation of anti-nociceptive inhibitory processes [133].

Maternal aerobic training also played an important role in cognitive decline prevention in Pb2+ exposed mice offspring [134].

Even forced exercise proved to be beneficial in association with topiramate (TPM) by potentiating its anti-seizure effect. It also inhibited the cognitive-impairment caused by TPM therapy [135].

Moderate treadmill exercise in mice can help prevent inflammatory events triggered by amyloid β (Aβ1–40) with regard to the NLRP3 inflammasome pathway in Alzheimer’s disease [136].

Nevertheless, given the pandemic period, it is worth mentioning that regular physical exercise proved to be useful in maintaining a strong immune function during the SARS-CoV-2 outbreaks [137].

However, recent data proved some deleterious myocardial effects of increased lifetime training hours (>6000 h) in recreational athletes. Left atrium dilation is an echocardiographic expression of atrial remodeling and it was significantly associated with the increased total training period. In fact, training duration proved to be an indirect and linear marker of left atrial volume and, therefore, a possible predictor factor for atrial fibrillation [138]. This increased arrhythmogenic risk is explained by the enhanced interstitial fibrosis and maladaptive structural changes secondary to pressure or volume overload, irrespective of the LV systolic function (which is usually preserved or even augmented in athletes). Inflammation and oxidative stress are also common aspects following intense training and are incriminated factors for atrial remodeling, as shown in a very recent review [139].

## 11. Conclusions

The study of biomarkers is a continually expanding medical domain and some novel laboratory explorations are rapidly gaining ground, not only in the cardiovascular pathologies, but also in the field of sports medicine. In this regard, emerging data may enable the practicians to routinely implement various new screening methods in order to facilitate the identification of individuals at-risk, either professional athletes or amateurs. There is still room for debate, given the limited available data, the extensive range of sports disciplines, training regimens, individual confounding factors and the lack of standardized protocols. Despite some very promising perspectives, extensive, more complex and more targeted studies are required to further confirm the role of biomarkers in the routine clinical practice related to sports medicine.

## Figures and Tables

**Table 1:** The classification of the biomarkers addressed in this review.

Immune	Endocrine	Inflammatory
HNP HBD IgA Leukocytes	IGF-1 Cortisol Testosterone	IL-6 TNF-α sTWEAK CD163 sST2

**Table 2:** Common causes of iron deficiency in athletes and their impact on physical performance and endurance.

Parameter	Etiology	Impact	References
Iron deficiency	Inadequate dietary intake Menstrual bleeding Impaired intestinal absorption due to effort-related inflammation Losses through erythrocyte destruction due to repeated foot striking in runners Losses through blood donation Losses through gastrointestinal bleeding due to increased consumption of non-steroidal anti-inflammatory drugs	Poor training endurance Difficult adaptation to high-altitude training Reduced energy efficiency Lower training volume/day Increased serum lactate Exhaustion occurs earlier Reduced myocardial performance	[54,119,120,121]
